# Dental public health: a trainee perspective from England

**DOI:** 10.1038/s41415-026-9966-2

**Published:** 2026-11-13

**Authors:** Anna Beaven, Samantha Watt, Ryan Grocock, Joelle Booth, Heather Raison

**Affiliations:** 573244540124091296335 School of Clinical Dentistry, University of Sheffield, Sheffield, UK; NHS England, England, United Kingdom; 951981882757029270140 NHS England, England, UK; University College London, London, United Kingdom; 396954601563677181858 NHS England, England, UK; 4Peninsula Dental School, University of Plymouth, Plymouth, United Kingdom; 414398106778049044274 NHS England, England, United Kingdom

## Abstract

Dental public health (DPH) specialty trainees are dentally qualified and focus on improving oral health at a population level in addition to individual patients. DPH trainees enter training with a broad range of dental career experiences. To attain specialist registration, DPH trainees undertake a 3–4-year programme which develops both technical and strategic leadership skills. DPH training includes activities such as advocating for oral health within the general health context, developing and applying evidence-based guidelines to support health improvement initiatives and addressing oral health inequalities amongst vulnerable and disadvantaged groups. Experiential learning is a key component of DPH training, delivered through projects and placements across a range of health and social care organisations. This trainee-authored paper provides an overview of the possible motivation for entering DPH specialty training, examples of the training experiences involved and consideration of how the wider dental workforce benefit from engaging with DPH teams.

## Key points

- Provides an overview of dental public health specialty training from four dentists who are either currently in training or have recently completed training in England.
- Describes the scope of specialty training activities, including key stakeholders and workstreams.
- Explains how readers may find opportunities to be involved with dental public health in their local areas.

## Introduction

### Overview of dental public health training

Dental public health is one of 13 dental specialties recognised by the UK General Dental Council (GDC). The specialty primarily focuses on improving population oral health outcomes and reducing oral health inequalities at a population level. Although dental public health consultants are not responsible for directly implementing service changes, they use several levers to drive improvement and innovation. This includes working with a wide range of organisations, systems and decision-makers to shape policy, inform commissioning, and influence service delivery, promoting equity in access, experience and outcomes. Specialty training prepares a trainee for the job role of a consultant in dental public health.

**Fig. 1:**
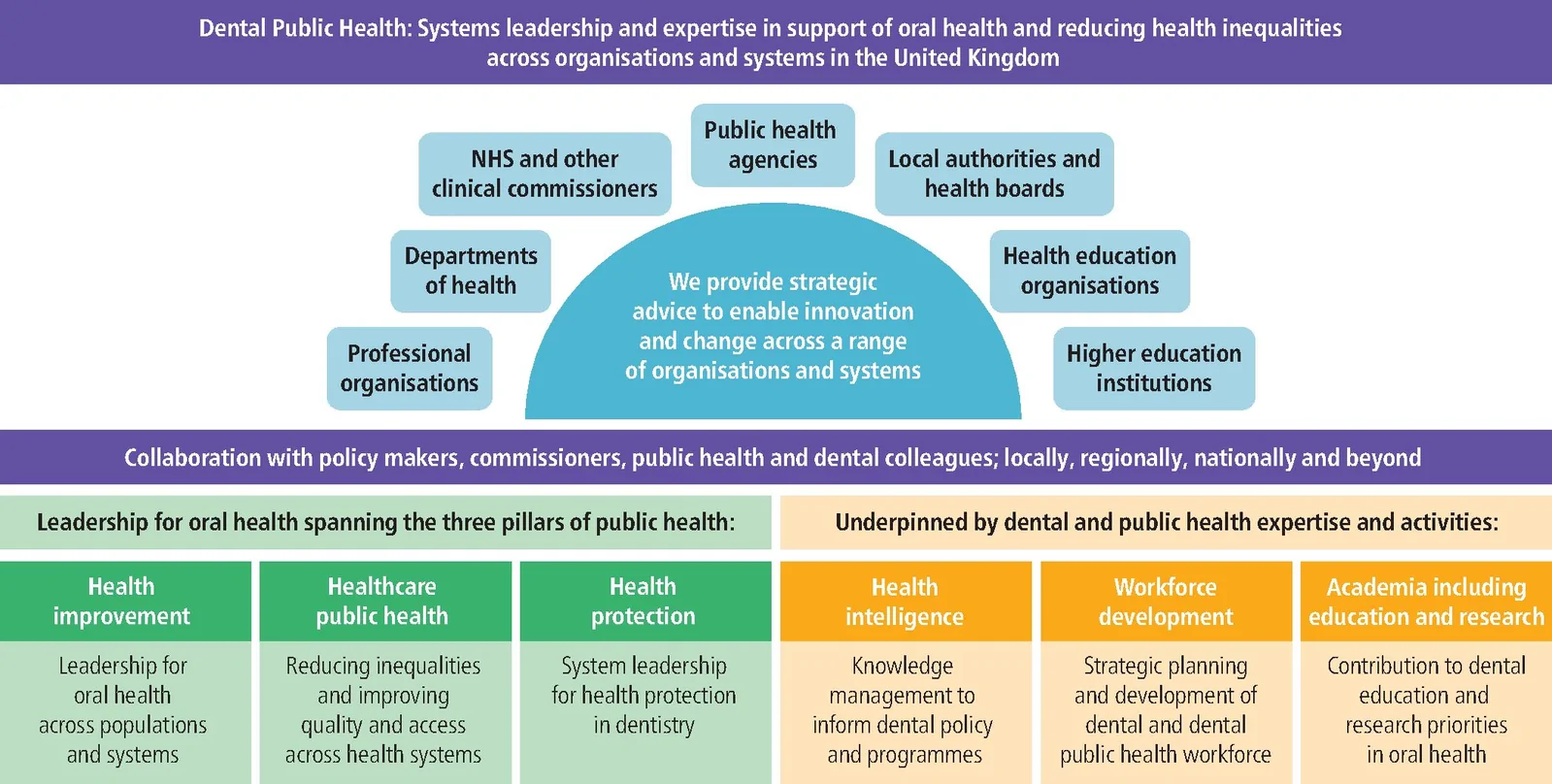
Reproduced with permission from, Gallagher J *et al*., Dental public health: connect, challenge and change, *Faculty Dent J* 2021; DOI: 10.1308/rcsfdj.202.22^2^

Under the GDC curriculum, dental public training is underpinned by the three pillars of public health: health improvement, healthcare public health and health protection.^1^ Dental public health expertise also involves health intelligence, workforce training and development and academia including research and education (Fig. 1).^2^

For the first year of training, a trainee will usually develop specialist-level knowledge and understanding of scientific concepts, theories and methods essential for dental public health practice. This involves completing a master's degree in dental public health, or public health with additional dental modules. The master's degree covers a number of core competencies relevant to dental public health practice such as statistics, epidemiology, health promotion and research methods. In addition, the dissertation component of the dental public health master's programme provides an opportunity to publish master's research findings in a peer-reviewed journal, representing a significant academic and professional achievement. Some trainees will enter the programme at year two if they already hold the relevant master's degree at the time of recruitment to the specialty.

From year two onwards, trainees working under consultant supervision apply their knowledge through experiential learning in dental public health practice. This involves activities such as assessing population health, critically appraising evidence-based advice and guidance, influencing oral health policy and providing strategic leadership support for commissioners across health and social care organisations, including government bodies.^2^ In England, trainees are hosted by health organisations such as the Office for Pan-ICB Commissioning. In the devolved nations, they are hosted by health boards in Scotland, the Public Health Agency in Northern Ireland, and Public Health Wales in Wales. Training is delivered through supervised project work under the guidance of a consultant in dental public health or public health. During specialty training, trainees may have worked closely with multiple consultants outside of their primary supervisor team. This can include consultants in other specialties and public health consultants.

In addition, some training posts, funded through the National Institute of Health Research (NIHR) also include an academic placement within a university. This offers the trainee the opportunity to develop research and teaching skills through contribution to dental education and university-specific oral health research priorities. The posts fit within the specialty training period and allow the trainee to combine both service and academic dental public health training. Academic Clinical Fellowships (ACFs) run for three years, with 75% of time allocated to service training and 25% allocated to academic training. The academic time is used to acquire academic skills and develop an application for a PhD (Doctor of Philosophy) fellowship, such as the NIHR doctoral award fellowship. Trainees who are successful in gaining PhD funding may complete this out of programme or less than full time (LTFT) in parallel to specialty training. If an individual already holds a PhD prior to specialty training, then they can apply for an academic clinical lectureship. This is a four-year combined programme, comprising 50% service training time and 50% academic training time to develop as an independent researcher and clinical academic leader. The training pathway is flexible. Non-academic service trainees may also apply for LTFT training or take time out of programme, whether this is for academic or non-academic reasons. Service trainees can also apply for doctoral fellowships and PhDs, with the caveat that they do not have the same amount of protected time as trainees doing an ACF.

There are multiple methods of assessing progression through training. These include academic exams, appraisals, work-based assessments, published reports, annual reviews of competence progression and specialty specific exams (delivered via the Royal College of Physicians and Surgeons in Glasgow). Once trainees pass all necessary components of training, they are awarded the Certificate of Completion of Specialty Training enabling them to join the GDC dental public health specialist list. Typically, dental public health consultant jobs will require the relevant master's degree, passing specialty-specific exit examinations, and the certificate of completion of specialty training.

### Application to specialist training

At the time of writing, there are 18 dental public health specialty trainees in the UK. Before entering the programme, trainees typically gain diverse clinical experience across specialties such as community dental services, special care dentistry, general practice, and oral and maxillofacial surgery. This varied background enhances understanding of the complexities within different dental specialties and service models. Such experience provides essential insight into the practicalities of service delivery and population needs which is critical for developing effective public health interventions and commissioning dental services. For example, one of the authors previously undertook a dental core training post in oral and maxillofacial surgery and community dental services. This developed her understanding of the process and limitations of epidemiology surveys, referral pathways for oral surgery, paediatrics and special care, and advocating for vulnerable populations.

As well as gaining experience in various branches of dentistry, there are specific activities that a potential applicant to dental public health training can seek out. These may include taking part in a service evaluation or quality improvement project within a clinical setting, joining one of the local managed clinical networks to understand system leadership for a specialty, involvement in local dental teaching, and contributing towards academic publications. We recommend contacting dental public health consultants and specialty trainees to understand more about the specialty and identify local opportunities to be involved with dental public health.

Motivation to enter specialty training varies among trainees (Box 1). However, a shared ambition unites them: to improve oral health outcomes at a population level and address oral health inequalities affecting society's most vulnerable populations. The application processes for DPH specialty training vary across the devolved nations in the UK. Scotland and Wales have local recruitment processes. In England and Northern Ireland, applications are through an annual national recruitment process. Applicants must have completed dental foundation training and dental core training (DCT) 1 and 2 or equivalent. Trainees beginning from 2025 must also have passed the Membership of the Faculty of Dental Surgery examinations to sit the final specialty examinations. The recruitment process for dental public health has an additional stage compared to other dental specialties to align recruitment with general public health training. This involves computer-based psychometric tests and is a requirement for eligibility for interview.^3^ These tests include numerical and critical reasoning and a situational judgement test. This provides one of the assurances for equivalency of training with general public health. After passing this test, applicants are invited to attend a national interview and rank available training posts.

Box 1 Trainee motivations for applying for dental public health **Why I entered dental public health specialty training**

- ‘Working in the community dental service, I saw the impact of poor oral health on children and the most vulnerable adults in society, even though tooth decay is nearly entirely preventable. I wanted to tackle this upstream'
- ‘On commencing a Dental Core Training post in the North East of England I saw that the north-south inequalities were stark, and I was saddened to see the extent of suffering of patients suffering from head and neck cancer and trauma. I attended a lecture at the Lindsay Society's annual conference in Bath, 2021. The talk covered public health approaches to violence prevention. I was inspired by this multi-sectoral, creative and data-driven approach. I wanted to learn more about dental public health after that!'
- ‘Working in many busy district general hospitals, I was acutely aware of the number of adults and children I would see with dental disease. They ended up in the emergency department, sometimes really unwell, and certainly in a lot of pain. I started exploring dental public health as a way of shaping services and targeting prevention to better serve the oral health needs of local populations'.

**Training highlight**

- ‘I was one of the lead authors of a national report on head and neck cancer, it's been amazing to see how the report has been used as a call to action to improve the vast inequalities we are currently seeing in head and neck cancers'
- ‘I have been working with health and justice commissioners to design pathways for dental care in secure settings and integrating oral health promotion into wider prison health-promoting activities'
- ‘Conducting a qualitative evaluation of a dental access initiative across an integrated care board area – focusing on patients' lived experiences and the impact of the pilot – was a standout experience and training highlight'.

## Dental public health trainee contributions to wider activities

Alongside the three pillars of public health – health improvement, healthcare public health and health protection – trainees in dental public health contribute to health intelligence, workforce development, academia and education. Examples of this include:

- Health intelligence – dental public health trainees are involved with collecting, interpreting and communicating data on the oral health needs of populations as well as mapping the provision of dental services alongside oral health improvement initiatives. Many dental public health trainee projects start by assessing the oral health needs of target populations. This is a vital step in informing the development of strategies to improve oral health and typically involves using data which have been transformed into public health intelligence. National examples include the National Dental Epidemiology Programme (NDEP) and National Oral Health Surveys, such as the Adult Oral Health Survey done in 2009 and 2023.^4^^,^^5^ These data are combined with locally collected intelligence through engagement with members of the public, dental professionals and commissioners
- Workforce development – within dental public health training many workstreams involve providing or facilitating education to dental professionals and health and social care professionals. Engagement with general dental practitioners is often facilitated through established networks, including the British Dental Association and NHS Workforce, Training and Education (NHS WTE). An example of this is a trainee project delivered alongside NHS WTE that focuses on improving the oral health of older adults living with dementia. Training on approaches to dementia-friendly dentistry was strategically delivered to dental teams, to address some of the challenges those living with dementia experience when visiting the dentist
- Academia, education and research – alongside clinical training, dental public health trainees are involved with research and education. Academically, many trainees contribute to the wider dental literature through publishing peer-reviewed articles showcasing oral health needs of populations, outlining oral health improvement strategies and documenting evaluations of oral health improvement interventions. Dental public health trainees may also deliver teaching and training to dental undergraduates, dental core trainees and non-dental professionals. As a trainee, this can be an enriching experience to spread awareness of the importance of dental public health, showcase what we do as a specialty and emphasise how we all have a role in improving the oral health of the populations with which we interact. An example from one of the authors was producing a scoping review, which was used directly to design the questionnaire in the 2024-2025 NDEP survey for adults aged over 65 living in residential settings.^6^^,^^7^

## Day in the life of a dental public health trainee – Anna Beaven

When I was considering applying for specialty training in dental public health, I would often ask the question to current trainees: ‘what does an average day look like?' The answer I often got in response was: ‘every day looks different!' Like all work, the job involves both exciting and routine elements. Specialty trainees need to meet their training competencies which are similar to clinical competencies in other specialties. They also have portfolio projects, which are like case presentations. All the training experiences contribute to a broader personal development which links to lifelong learning as a dental professional.

During my training, I am split between working from home, in the office and at face-to-face meetings and workshops. Working from home or in the office may include attending meetings such as integrated care board commissioning meetings, local dental networks, and networking with other organisations, such as regional inclusion health public health meetings. These offer opportunities to influence what work is being done around dentistry with the aim of promoting oral health and reducing inequalities. Desktop work may include responding to enquiries, preparing papers (such as briefing papers or guidance), or reflecting on training and monitoring my progress. However, the role of dental public health is not simply to attend these meetings. These provide an opportunity to scope the nature of a situation or problem; to consider what solutions may be feasible and appropriate; and to influence stakeholders within a particular setting, through relationship-building. Dental public health is both a science and an art, meaning that there is not always one correct way to approach a situation. This depends on a combination of factors, requiring a perceptive mind, and there is scope for creativity to one's approach.

In-person days also vary significantly. Relationships are key to the role of a dental public health practitioner. One may be attempting to influence the health of a population by working with people who have multiple competing priorities. Being able to identify opportunities where goals align, nurturing relationships with stakeholders, and scoping opportunities for innovation, is hugely important to the role of a dental public health practitioner.

Another question that outsiders to the specialty may ask is: ‘does any of this office-based work have an actual impact?' I would like to share an example of some impacts from my work oriented towards health inequalities. I have been working on projects to develop the dental workforce to support those experiencing multiple unmet needs, who are socially excluded.^8^ This includes bringing together a group of representatives from local authorities, voluntary, community and nonprofit organisations, integrated care boards, dental commissioners, and dental team members. I led a service evaluation on dental services for inclusion health groups which I then used to advocate for continued funding for these services. Multi-agency partnership working is a huge element of dental public health practice, and being able to ensure that any workstreams are completed in collaboration with a range of stakeholders allows the best chances of success. This work has been recognised in regional and national networks as a best practice example of reducing inequalities in access to primary care services. An example of a typical dental public health training week is shown in Table 1.

**Table 1:** Exemplar timetable for dental public health trainee with examples

**Monday**	**Tuesday**	**Wednesday**	**Thursday**	**Friday**
**Trainee tutorial** This could be with a local dental public health consultant and may involve discussion of an over-the-desk challenge that a consultant may have recently faced.	**Work-based assessment with ES** Similar to assessments done in DFT and DCT, these will typically include evaluation of an activity and a reflective discussion. Early in training this could include simple tasks such as chairing a meeting or producing a briefing paper; later in training it could include discussing what you would do in a given scenario.	**Visit local site** Getting ‘on-the ground' experience of visiting your stakeholders allows you to better understand how a project might be working. Face to face conversations can also allow you to understand the motivations, attitudes and challenges an organisation may be facing, so you better know how to approach a situation. Visiting local sites can develop insights, such as how a local dental practice is engaging with outreach for people experiencing homelessness.	**Desktop work – data** Desktop work varies hugely, but sometimes it involves reviewing data and interpreting it to ‘tell a story'. This is key evidence-based practice. Examples could include supporting local authorities with sampling for epidemiology in alignment with national dental public health guidelines, or evaluating contract data as part of a service evaluation. Data might also be processed when preparing manuscripts for publication.	**Public health workshop – co-production, inclusion health** There are educational opportunities aligned with public health registrars including registrar meetings, tutorials, and workshops. This workshop looks at listening to the voices of socially excluded groups to ensure that public health activities best meet the needs of these communities.
**Desktop time, emails, reflecting, e-portfolio, project planning** As a trainee, you will have multiple e-portfolio activities to complete, similar to DFT and DCT. Emails vary widely in scope and can be as simple as bringing stakeholders together to arrange a meeting, reaching out to new people, and keeping up to date with the wider public health landscape.	**Meetings – local dental network, managed clinical network, ICB commissioning governance meetings** You may be attending a local governance meeting to understand more about the commissioning landscape or to scope existing inequalities and opportunities for dental public health to contribute. I have presented ideas at these meetings to seek feedback and endorsement from clinicians and commissioners.	**Local authority meetings** This could include a local authority oral health advisory group. On placement this may include meeting local councillors with the portfolio for health, attending Health and Wellbeing Board, or Scrutiny Committees.	**Biweekly team meeting** Team meetings are key elements of dental public health training. They allow learning to be shared between consultants and trainees, for issues and priorities to be identified, and for support to be given with training or other workstreams.	**Desktop time, meeting stakeholders in projects – dentists, commissioners, MCN chairs, other public health colleagues** Talking to people is one of the most important activities of our job role. Meeting stakeholders can be simple introductions to build your network, a chance to troubleshoot an issue, and to brainstorm or bring people on board with your ideas.
DCT, Dental Core Training; DFT, Dental Foundation Training; ES, Educational Supervisor; ICB, Integrated Care Board; MCN, Managed Clinical Network.				

## How can/should the wider dental workforce become involved?

There are many opportunities to learn more about dental public health and upskill. Some regions offer training posts at dental foundation and dental core training level where dental public health placements are offered alongside clinical training. This introduces dental public health to both develop skills that can be applied to all dental career paths and whether a career in dental public health is something you wish to explore further. Outside of formalised training roles, some organisations, such as the community dental services have oral health improvement roles. These providers work closely with dental public health to operationalise oral health improvement and epidemiological programmes including supervised toothbrushing and the NDEP. In addition, UK academic institutions offer master's level qualifications in dental public health. There are also many short courses, such as on ‘e-learning for healthcare',^9^ on prevention for the whole dental team and wider health and social care professionals. Topics include: children's oral health, mouthcare for those in hospital settings, and Making Every Contact Count training for smoking and alcohol consumption. There are also a variety of non-clinical working opportunities such as the Faculty of Medical Leadership and Management Chief Dental Officer Clinical Fellow scheme, which gives exposure to systems leadership and understanding how policy is developed and implemented.

The specialist society for dental public health is the British Association for the Study of Community Dentistry (BASCD). This is UK's professional association for the science, philosophy and practice of promoting oral health in society. While many of those working in dental public health are members of BASCD, many other individuals with an interest in improving oral health are members including dentists, dental care professionals, public health practitioners and academics. Regular scientific conferences, professional development opportunities and working groups are held to bring those together with an interest in population oral health to collaborate, share best practice, and contribute to national policy and strategy. Joining BASCD is also a way of finding out more about the specialty. The European Association of Dental Public Health is another association that trainees, specialists, and those interested in dental public health may be part of. They host regular webinars and an annual conference, which provides an opportunity to network, collaborate, and learn about dental public health across Europe.

## Conclusion

Dental public health training encompasses both theoretical and applied skills development in strategic leadership to promote and protect oral health and reduce inequalities. Supported by collaborative networks and embedded within the wider health and social care system, dental public health training offers a dynamic, non-linear, yet purposeful career path, one which presents new challenges and opportunities daily. Hopefully after reading this article, you will feel either inspired to join the training programme yourself, or to seek opportunities to support public health initiatives within your local community. Dental public health trainees are a friendly group. The reader is welcome to contact the authors to learn more about the specialty or to be put in touch with their local networks.
